# Engineering a conduction‐consistent cardiac patch with graphene oxide modified butterfly wings and human pluripotent stem cell‐derived cardiomyocytes

**DOI:** 10.1002/btm2.10522

**Published:** 2023-04-14

**Authors:** Yao Tan, Tingting Lu, Ying Chen, Nevin Witman, Bingqian Yan, Li Yang, Minglu Liu, Yiqi Gong, Xuefeng Ai, Runjiao Luo, Huijing Wang, Wei Wang, Wei Fu

**Affiliations:** ^1^ Institute of Pediatric Translational Medicine Shanghai Children's Medical Center, School of Medicine, Shanghai Jiao Tong University Shanghai China; ^2^ Department of Clinical Neuroscience Karolinska Institutet Stockholm Sweden; ^3^ Department of Anesthesiology Fudan University Shanghai Cancer Center Shanghai China; ^4^ Department of Oncology Shanghai Medical College, Fudan University Shanghai China; ^5^ Department of Pediatric Cardiothoracic Surgery Shanghai Children's Medical Center, School of Medicine, Shanghai Jiao Tong University Shanghai China; ^6^ Shanghai Key Laboratory of Tissue Engineering Shanghai 9th People's Hospital, School of Medicine, Shanghai Jiao Tong University Shanghai China

**Keywords:** cardiomyocyte maturation, conduction consistent, induced pluripotent stem cells, morpho butterflies, oxidized graphene

## Abstract

Engineering a conduction‐consistent cardiac patch has direct implications to biomedical research. However, there is difficulty in obtaining and maintaining a system that allows researchers to study physiologically relevant cardiac development, maturation, and drug screening due to the issues around inconsistent contractions of cardiomyocytes. Butterfly wings have special nanostructures arranged in parallel, which could help generate the alignment of cardiomyocytes to better mimic the natural heart tissue structure. Here, we construct a conduction‐consistent human cardiac muscle patch by assembling human induced pluripotent stem cell‐derived cardiomyocytes (hiPSC‐CMs) on graphene oxide (GO) modified butterfly wings. We also show this system functions as a versatile model to study human cardiomyogenesis by assembling human induced pluripotent stem cell‐derived cardiac progenitor cells (hiPSC‐CPCs) on the GO modified butterfly wings. The GO modified butterfly wing platform facilitated the parallel orientation of hiPSC‐CMs, enhanced relative maturation as well as improved conduction consistency of the cardiomyocytes. In addition, GO modified butterfly wings enhanced the proliferation and maturation characteristics of the hiPSC‐CPCs. In accordance with data obtained from RNA‐sequencing and gene signatures, assembling hiPSC‐CPCs on GO modified butterfly wings stimulated the differentiation of the progenitors into relatively mature hiPSC‐CMs. These characteristics and capabilities of GO modified butterfly wings make them an ideal platform for heart research and drug screening.

## 1 INTRODUCTION

The surfaces of butterfly wings have special nanostructures arranged in parallel which can generate cellular alignment; these structures can also be electro‐mechanically manipulated in order to form vivid structural colors.^1^, ^2^, ^3^, ^4^, ^5^, ^6^ Although butterfly wings have some individual differences and potential issues with nature conservation, the current level of artificial technology does not simulate such a suitable structure.^1^, ^7^ Previous studies have shown that these nanostructures induced neonatal rat ventricular cardiomyocytes to align in the same orientation, allowing the researchers to develop a biosensor that reflected the beating of cardiomyocytes through the changes in the structural color of butterfly wings.^1^ Rodent ventricular cardiomyocytes are not ideal for studying human cardiac diseases and the acquisition of induced pluripotent stem cells (iPSCs) has helped to overcome ethical issues related to obtaining cardiomyocytes from embryonic stem cells, while simultaneously providing a never‐ending source of human cardiac cells.^8^, ^9^

The reprogramming of somatic cells into iPSCs and the directed differentiation of iPSCs to cardiomyocytes has led to a personalized method for cardiovascular disease research and drug screening. Furthermore, in vitro differentiation processes can be employed to study, for example, the cellular and molecular mechanisms of cardiac development and the pathogenesis of congenital heart diseases.^10^, ^11^, ^12^ However, several limitations and hurdles exist for the generation of functionally mature iPSC‐CMs for regenerative therapy, cardiac drug screening, or cardiac development research. Many 2‐D culture systems generate cardiomyocytes derived from iPSCs in a state with disordered and spontaneous contraction, which inaccurately simulates the consistent and holistic beating of myocytes in vivo.^13^ In addition, current methodologies that generate iPSC‐CMs produce structurally underdeveloped and immature cardiomyocytes.^14^ Our previous study showed that seeding iPSCs into a microgroove structural gelatin film was conducive to the differentiation of relatively more mature cardiomyocytes.^15^ We speculated that increasing cell conduction consistency based on the special grooved structure together with the application of a conductive substance could further promote the process of hiPSCs differentiating into cardiomyocytes.

Graphene oxide (GO) is a promising conductive material for heart tissue engineering scaffolds.^16^ GO has been shown to promote cellular adhesion, growth, and proliferation due to favorable mechanical and electrical properties, strong surface chemistry, and special antibacterial properties.^16^, ^17^, ^18^, ^19^ Previous reports have shown that the application of GO can simultaneously improve the mechanical properties and electrical conductivity of gelatin methacryloyl (GelMA) hydrogels.^20^ In addition, studies have shown that GO can promote stem cell differentiation into ectoderm, mesoderm, and endoderm lineages, such as bone cells, nerve cells, and blood cells.^21^, ^22^, ^23^, ^24^, ^25^ Interestingly, previous studies revealed that GO promoted the functional maturation of neonatal rat cardiomyocytes.^26^, ^27^ The adhesion of the plasma membrane to GO and the cytoplasmic accumulation of GO may accelerate the early stages of iPSC‐derived cardiac differentiation.^25^

**FIGURE 1:**
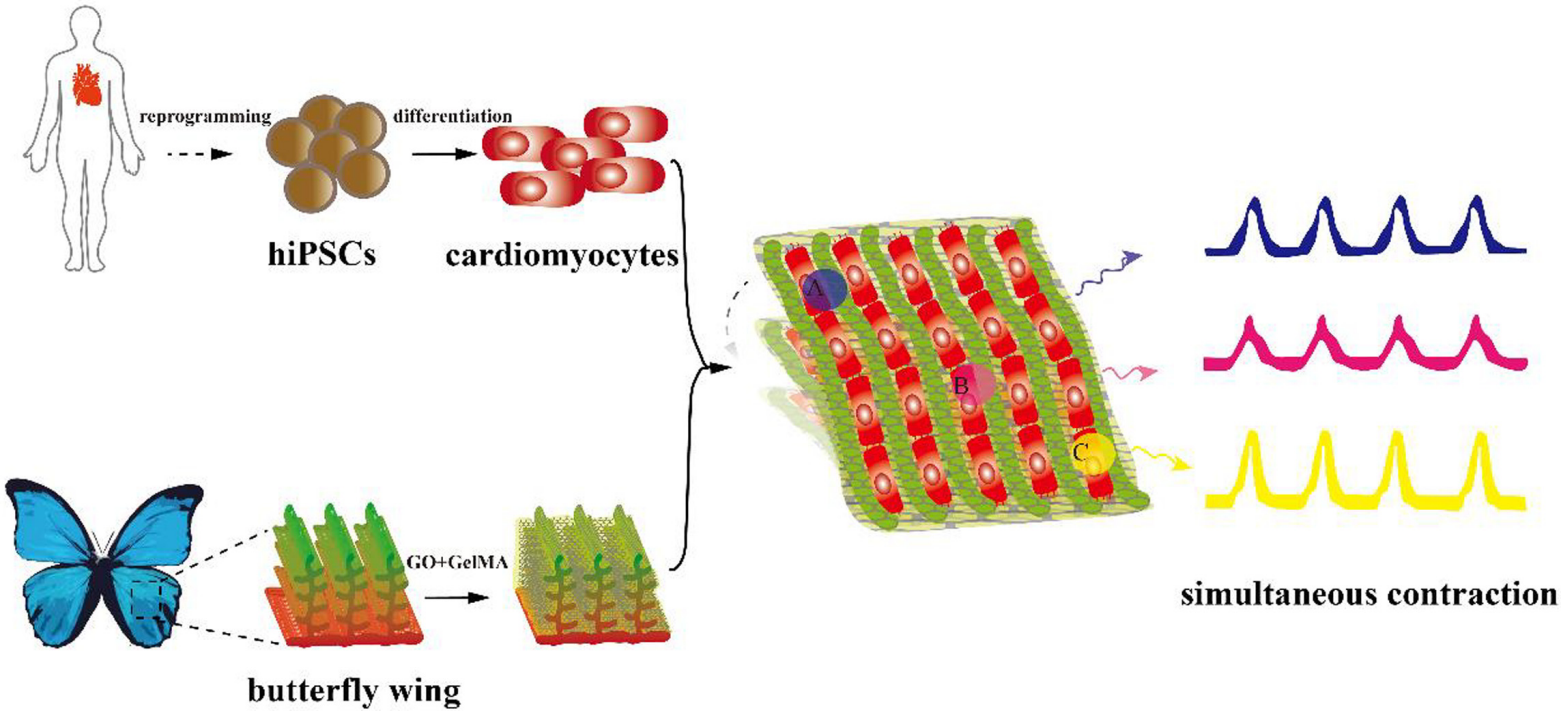
Scheme of the construction of the simultaneous contraction platform by assembling hiPSC‐CMs on the GO‐modified butterfly wings.

Herein we show that GO can be employed to modify butterfly wings in order to enhance the regulation of human cardiomyocyte conductivity through the construction of a conduction‐consistent physiological cardiac muscle patch (Figure 1). Additionally, hiPSC‐CPCs were used to verify that electrical conduction consistency can promote the differentiation of stem cells toward cardiomyocytes, to construct a cardiac patch that can simulate the differentiation and development of cardiomyocytes for drug screening, disease modeling, and additional research.

## 2 MATERIALS AND METHODS

### 2.1 Preparation of GO modified butterfly wings

We purchased the Morpho Menelaus (M. Menelaus) butterflies from Shanghai Qiuyu Biological Technology Co. To increase the butterfly wings' hydrophilicity and biocompatibility, we added 5% (w/v) GelMA to them. GelMA is a semi‐synthetic hydrogel which consists of gelatin derivatized with methacrylamide and methacrylate groups. We then made a further refinement of butterfly wings through mixing 0.1% (w/v) graphene oxide (GO) in GelMA to obtain GO modified butterfly wings, to increase the conductivity of butterfly wings.

### 2.2 Characterizations of GO modified butterfly wings

The microstructure and surface morphology of three kinds of butterfly wings were studied using scanning electron microscopy (SEM). After spraying gold on the surface of the samples, the photos were taken under SEM. We selected 100 points for width statistics. The contact angles of butterfly wings were measured by the JY‐82 contact machine, the samples were loaded on the stage of contact angle tester and 12 μL distilled water was placed on the samples. The contact angle was measured by large ellipse method. Three samples were tested in each group.

### 2.3 Differentiation and cultivation of hiPSC‐CPCs and hiPSC‐CMs

hiPSCs from healthy male newborns' foreskin cells were kindly provided by Stem Cell Bank, Chinese Academy of Science. Both cardiac progenitor cells and cardiomyocytes were differentiated from human induced pluripotent stem cells (hiPSCs). The hiPSCs were self‐prepared and maintained in TeSR‐E8 (5990, STEMCELL, CA). Detachment of cells from the six‐well plates was performed when the cells reached 80%–90% confluency using Accutase (7920, STEMCELL, CA). After centrifuging for 5 min at 1000 rpm, cells were resuspended in TeSR‐E8 and mixed with 5 μM Y‐27632 (72304, STEMCELL, CA) for continued culture in constant temperature (37°C) and humidity incubator with 5% CO_2_. Forty‐eight hours post seeding, the TeSR‐E8 media is replaced with differentiation medium I with 12 μM CHIR99021 (72054, STEMCELL, CA). Preparation of the differentiation medium I was performed by mixing 500 mL RPMI1640 (11875093, Life Technologies, USA) and 10 mL B27 supplement minus insulin (A1895601, Life Technologies, USA). Thirty‐six hours later, the medium is replaced with fresh differentiation medium I. One day later, media was changed with differentiation medium I mixed with 5 μM IWP‐2 (72124, STEMCELL, CA). Subsequently culture medium was replaced with differentiation medium I 2 days later. On Day 6, we obtained hiPSC‐CPCs which were collected for additional trials (see below). On Day 7, the culture medium was replaced by the differentiation medium II which is made by mixing 10 mL B27 supplement (17504044, Life Technologies, USA) with 500 mL RPMI1640. Culture medium is replaced with the differentiation medium II every 2 days. By Day 15, beating hiPSC‐CMs are observed.

For hiPSC‐CPCs studies on butterfly wings, the hiPSCs differentiated to the 6th day were digested with Accutase for 15 min after washed by PBS once, then the cells were resuspended in the differentiation medium I with 5 μM Y‐27632 after centrifugation at 1000 rpm. For hiPSC‐CMs studies on butterfly wings, the hiPSCs differentiated to the 15th day were washed with PBS once, and digested with myocardial cell digestive solution I (CA2011100, Cellapy, CN) for 10 min, then digested 20 min with myocardial cell digestive solution II (CA2012100, Cellapy, CN) and centrifuged at 1000 rpm. The cells were then resuspended in the differentiation medium II with 5 μM Y‐27632.

The GO and GelMA modified butterfly wings were cut into the 24‐well‐size and maintained in 24‐well plates soaked in 75% ethyl alcohol for 5 min and then exposed to ultraviolet radiation for another 30 min for sterilization purposes. The butterfly wings are suspended in either hiPSC‐CPCs solution or hiPSC‐CMs solution and cultured at constant temperature (37°C) and humidity incubator with 5% CO_2_.

### 2.4 Immunostaining studies

The samples were washed with PBS (SH30256.01, Hyclon, USA) once to remove dead cells. Then they were immersed in 4% v/v paraformaldehyde (P0099, Beyotime, CN)/PBS solution and 0.25% v/v Triton‐X100 (MB2486‐1, Meilunbio, CN)/PBS solution for 30 min to immobilize protein and permeabilize cytomembrane respectively. Five percent BSA (A3828‐100, MultiSciences [Lianke] Biotech, CN)/PBS was used for immunofluorescence blocking. Incubation of the primary antibody pluripotent markers Nanog (ab 109250, Abcam, GB), Sox2 (ab92494, Abcam, GB), Oct4 (ab181557, Abcam, GB), TRA‐1‐60 (ab16288, Abcam, GB)/ISL1 (39.3f7, DSHB, USA)/α‐actin (127M4807, Sigma, DE) and cTnT (15513‐1‐AP, Proteintech, USA) or CX43 (ab11370, Abcam, GB) was performed by 1:200 dilution for hiPSCs/hiPSC‐CPCs/hiPSC‐CMs overnight respectively at 4°C, followed by labeling with the fluorescent secondary antibody (1:1000 dilution) and incubated for 2 h at room temperature. Nuclear counterstain was performed by DAPI (40728ES03, Yeasen, CN) in 1:1000 dilution for 5 min at room temperature. Laser confocal microscope (Leica, TCS SP8, AT) was used for observation and photo recording.

### 2.5 Transmission electron microscopy

We used transmission electron microscopy (TEM) to study the microstructure of hiPSC‐CMs. The cardiomyocytes differentiated to the 25th day were fixed with 2.5% glutaraldehyde in PBS at 4°C for 2 h. Cells were washed with PBS twice at 4°C, 10 min for each time. Next, cells were post‐fixed in 1% osmium tetroxide at 4°C for 2 h. Double distilled water was used to wash the cells at 4°C for 10 min twice. The cells were then dehydrated by a graded ethanol series (30%–50%–70%–80%–95%–100%–100%), where each graded ethanol consumed 10 min. During dehydration, we used 3% uranyl acetate in 70% ethanol to stain the cells. After dehydration, cells were embedded in Epon 812. Ultrathin sections were cut with a ultramicrotome (LEICA, EM UC7, AT) and stained with lead citrate for further analysis by electron microscopy (HITACHI, H‐7650, JP). Images were obtained with technical help from the team of public application platform for basic medicine of Shanghai Jiaotong University School of Medicine.

### 2.6 Patch clamp

To record the cellular action potentials, hiPSC‐CMs were reseeded on a glass bottom Petri dish (801002, NEST, CN). The dish was mounted onto the stage of an inverted microscope (Ti–U, Nikon, JP) equipped with a patch clamp amplifier (MultiClamp700B, Axon CNS, USA). Whole‐cell patch clamp was performed on the cells at 36–37°C using a sharp microelectrode with a tip resistance between 3 and 5 MΩ which was made by using a micropipette piller (P97, Shutter Instrument, USA) to pulling a standard‐wall borosilicate glass capillary tube (B15023F, VitalSense, CN). The action potential inner solution was composed of 2.0 mM MgCl_2_, 150 mM KCl, 2.0 mM Na_2_ATP, 5.0 mM EGTA and 10 mM HEPES, and pH was adjusted to 7.2 with Tris. The action potential outer solution was composed of 1.0 mM MgCl_2_, 2.0 mM CaCl_2_, 5.0 mM KCl, 130 mM NaCl, 10 mM sucrose, 10 mM HEPES and 20 mM glucose, and pH was adjusted to 7.4 with Tris.

### 2.7 Flow cytometry

Foxp3/transcription factor staining buffer set (00‐5523‐00, eBioscience, USA) was used for immobilizing protein and permeabilizing the cytomembrane according to the manufacturer's instructions. First, the fixation/permeabilization concentrate was mixed with fixation/permeabilization at a ratio of 1:3 to prepare the working fixation/permeabilization solution. The permeabilization buffer was prepared by diluting permeabilization buffer with distilled water at a ratio of 1:9. The hiPSC‐CPCs/hiPSC‐CMs were immersed with 200 μL working fixation/permeabilization solution and incubated on ice for 30 min. Primary antibody staining was performed using ISL1/cTnT at 1:200 and left to incubate on ice for 30 min. Afterwards, the corresponding fluorescent secondary antibody was diluted to 1:1000 and left to incubate for 30 min and then centrifuged the cell suspension at 1000 rpm. Remove the supernatant and resuspended the cells with 300 μL permeabilization buffer for the further flow cytometry analyze.

### 2.8 Calcium imaging

hiPSC‐CMs were incubated in indicator‐free 1640 (11835030, Life Technologies, USA) with 5 μM rhod2 (R14220, Life Technologies, USA) for 60 min at 37°C. Before fluorescence measurements, cells were washed in indicator‐free 1640, and then incubated in it for a further 30 min. Afterwards, spontaneous increase in intracellular Ca^2+^ concentration was imaged using a fluorescent microscope (Leica, TCS SP8, AT) at 561 nm wavelength. Substrates were connected to a pacemaker (YC‐3 Bipolar Programmable Electrical Stimulator, CHINA) and paced 10 ms, 5 V/cm, 1 Hz. The experiment was repeated three times.

### 2.9 EDU assay

After seeding hiPSC‐CPCs on GelMA and GO modified butterfly wings, we used the EDU kit (c0078s, Beyotime, CN) to detect the proliferation of hiPSC‐CPCs at early (D7‐D8) and late (D12‐D13) stages of the differentiation protocol. We replaced the culture medium with differentiation medium II with 10 mM EDU on Day 7 and Day 12 of differentiation. Twenty‐four hours later, the EDU click reaction solution was added after these cells were fixed by 4% v/v paraformaldehyde/PBS and had been permeabilized by 0.25% v/v Triton‐X100/PBS. After incubating the cells in the dark at room temperature for 30 min, we used DAPI (1:1000 dilution) to perform the nuclear counterstain. Laser confocal microscopy was used for observation and photo recording.

### 2.10 Bulk RNA‐seq

We use hiPSC‐CPCs (Day 6), early state hiPSC‐CMs (Day 10) and late state hiPSC‐CMs (Day 15) to explore the effects of GO on the differentiation of hiPSC‐CPCs into hiPSC‐CMs. Trizol was used to lyse the collected cells. The RNA was extracted with isopropanol, and the concentration and quality of total RNA were detected by nanodrop 2000. The cDNA was synthesized by reverse transcription of the extracted RNA. After terminal repair and linker ligation, the product was amplified and cyclized to establish a library. The size and concentration of the library fragment were detected by Agilent 2100 Bioanalyzer, and the sequence was performed by combined probe anchored polymerization (CPAs). Quality control of the data was carried out by Soapnuke, and the gene expression level was calculated by RSEM. Deseq2 was used to analyze the differentially expressed genes. GO and KEGG were used to enrich the differentially expressed genes. The function and related pathways of the differentially expressed genes were analyzed.

### 2.11 Data analysis

Image J was used to randomly select 440 data points in photos of hiPSC‐CMs on butterfly wings to measure the angle distribution of fiber, and statistical analysis was carried out to draw the angle distribution map and the ratio of cell length to width. The expression of Cx43 was also analyzed by image J. The calcium imaging traces were analyzed by Leica Application Suite X 3.5.6. FlowJo was used to analyze the results of flow cytometry.

### 2.12 Statistical analysis

We used unpaired Student's *t*‐tests with GraphPad Prism software to evaluate all statistical analysis. Data are all presented as mean ± SD, and *p* < 0.05 is considered significant.

## 3 RESULTS

### 3.1 Fabrication and characteristics of GO modified butterfly wings

**FIGURE 2:**
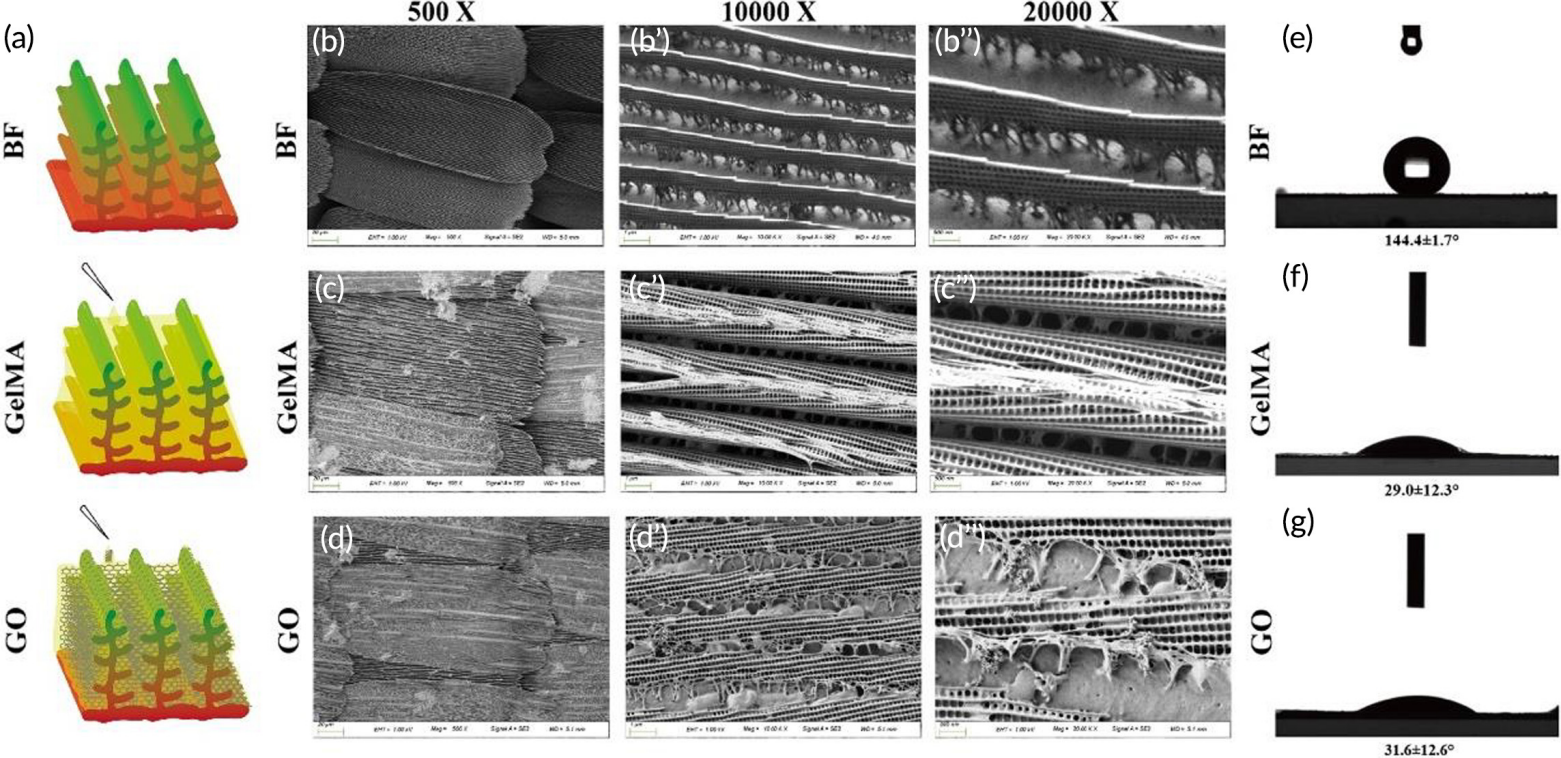
Fabrication and characteristics of GelMA and GO modified butterfly wings. (a) The schematic diagram of the fabrication of the GO modified butterfly wing. (b–b″) The SEM image of the single butterfly wing (BF) with different magnification. (b) Scale bar, 20 μm. (b′) Scale bar, 1 μm. (b″) Scale bar, 500 nm. (c–c″) The SEM image of the single GelMA modified butterfly wing (GelMA) with different magnification. (c) Scale bar, 20 μm. (c′) Scale bar, 1 μm. (c″) Scale bar, 500 nm. (d–d″) The SEM image of the single GO modified butterfly wing (GO) with different magnification. (d) Scale bar, 20 μm. (d′) Scale bar, 1 μm. (d″) Scale bar, 500 nm. (e–g) The contact angle of the BF, GelMA, and GO.

We selected butterfly wings as a base scaffold for the enhancement of conduction and maturation of cardiomyocytes, as they contain periodic parallel nanoridges that can guide cell arrangement.^1^, ^28^, ^29^ As the hydrophilicity of simple butterfly wings is weak, we added 5% (w/v) GelMA (GelMA modified butterfly wings), a semi‐synthetic hydrogel which consists of gelatin derivatized with methacrylamide and methacrylate groups to increase their hydrophilicity and biocompatibility properties. To further increase the conductivity of the culture substrate, we mixed the 0.1% (w/v) graphene oxide (GO) into GelMA to further modify the butterfly wings (GO modified butterfly wings) (Figure 2a). Imaging analysis of the single, GelMA, and GO modified butterfly wings was taken using scanning electron microscopy (SEM), which revealed the periodic parallel nanoridges. The groove between the nanoridges was about 0.5–1.5 μm and the size of each single butterfly wing was about 0.02 mm^2^ (Figure 2b–d). For reference, this size would enable the cellular seeding of 1–2 mature adult ventricular myocytes.^30^ On the surface of the modified butterfly wing, there were some discontinuous shapes between the ridges of scales because the GelMA packed the nanoridges together to some extent, yet the whole scale revealed obvious groove structures with certain arrangement directionality (Figure 2c′–d′,c″–d″). The contact angle of the butterfly wing was 144.4 ± 1.7° which showed the weak hydrophilicity (Figure 2e). After GelMA was added, the contact angle decreased to 29.0 ± 12.3° and 31.6 ± 12.6°, an indication that GelMA significantly improved the hydrophilicity of the butterfly wing (Figure 2f). The contact angle did not significantly change with the addition of GO (Figure 2g).

### 3.2 Differentiation and characteristics of the hiPSC‐CMs

**FIGURE 3:**
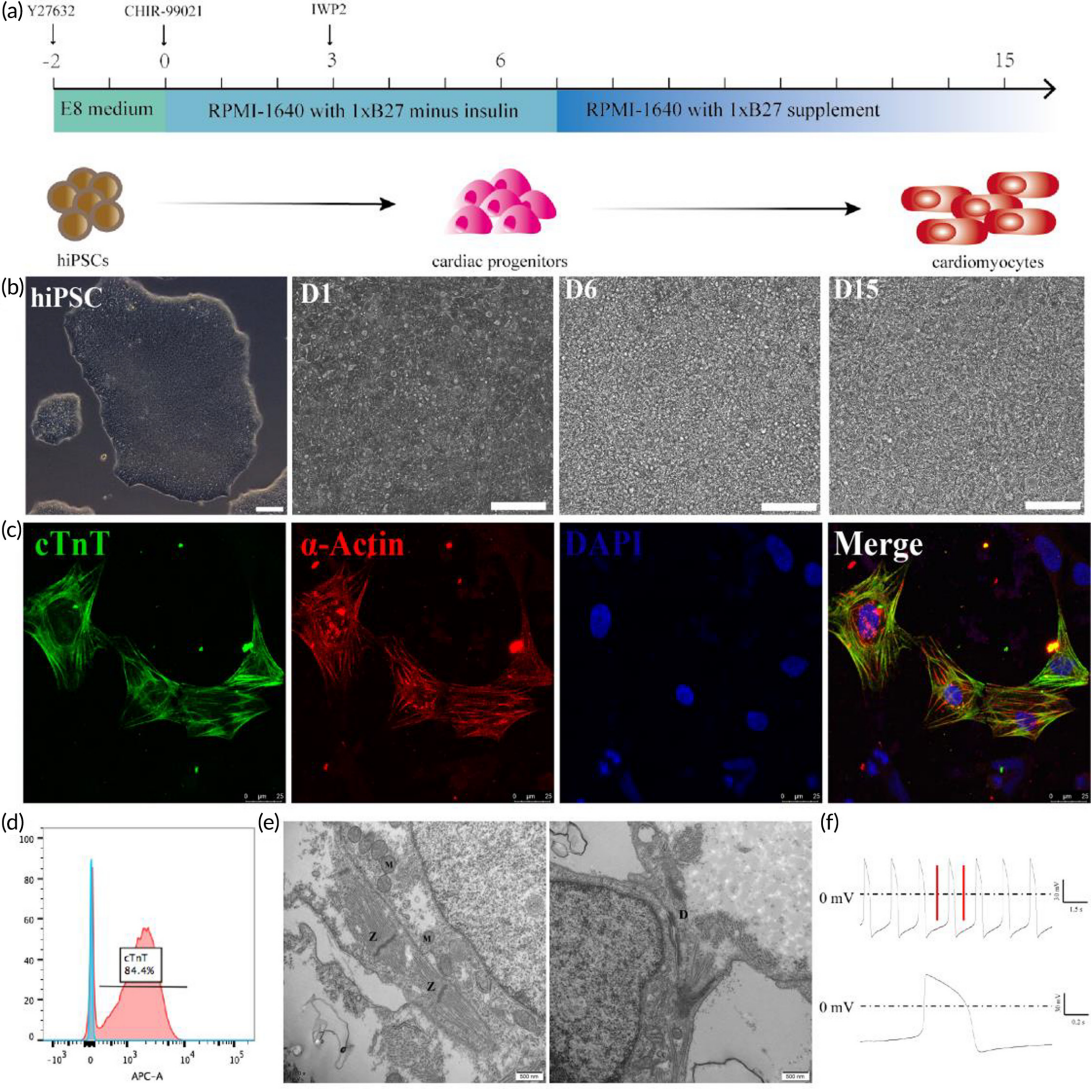
Molecular and functional validation of hiPSC‐CM generation in culture plates. (a) Schematic diagram of the cardiomyocytes differentiation from hiPSCs. (b) Brightfield of hiPSCs differentiation; from left to right are: before induction of differentiation, scale bar, 200 μm; cells on differentiation Day 1, scale bar, 100 μm; hiPSC‐CPCs on differentiation Day 6, scale bar, 100 μm; hiPSC‐CMs on differentiation Day 15, scale bar, 100 μm. (c) The immunofluorescence staining of the hiPSC‐CMs, scale bar, 25 μm. cTnT (green), α‐actin (red), and nucleus (DAPI). (d) Flow cytometry of cTnT in the hiPSC‐CMs on Day 15. (e) TEM image of the hiPSC‐CMs, scale bar 500 nm. Z line (Z), mitochondrion (M), intercalated disk (D). (f) Typical action potential of an individual hiPSC‐CM recorded via patch clamp.

Next, we aimed to generate hiPSC‐CMs for downstream seeding experiments on the GO modified butterfly wings, by first validating our approach on plates. First, we verified that hiPSCs from our working banks maintain pluripotency throughout entirety of the experiment by visualizing the continual expression of stem cell markers OCT4, TRA‐1‐60, SOX2, NANONG (Figure S1). To generate cardiac derivates for all studies conducted, we employed iPSCs between passages 30 and 50. Cardiomyocyte differentiations from hiPSCs were induced using CHIR99021 and IWP‐2 according to a previously published protocol (Figure 3a).^9^ Cardiomyocytes were successfully generated with a phenotypic characteristic of autonomous beating on approximately the 10th day of differentiation (Video S1). The differentiations were carefully monitored and by the 15th day of the differentiation protocol we verified and characterized the expression of the well‐known cardiomyocyte marker genes, α‐actin, and cTnT (Figure 3b,c). In addition to these structural observations, flow cytometry analyses indicated consistently high and efficient differentiations (80%–90% cTnT+) by Day 15 of the protocol (Figure 3d). The cellular ultrastructure of hiPSC‐CMs of Day 15 was visualized by TEM. The results showed that the hiPSC‐CMs possess abundant myofibrils, and the z‐lines are clearly visible. Several mitochondria and intercalated disks can be seen in the cytoplasm and between cells, respectively (Figure 3e). Action potentials of the hiPSC‐CMs were also recorded using patch clamp techniques. The patch‐clamp recordings of the spontaneously beating cardiomyocytes demonstrated that the action potentials were ventricular‐like, which agrees with the classification criteria of hiPSC‐CMs, as previously reported (Figure 3f).^31^

### 3.3 GO modified butterfly wings guide the directional alignment of hiPSC‐CMs and promote their maturation

**FIGURE 4:**
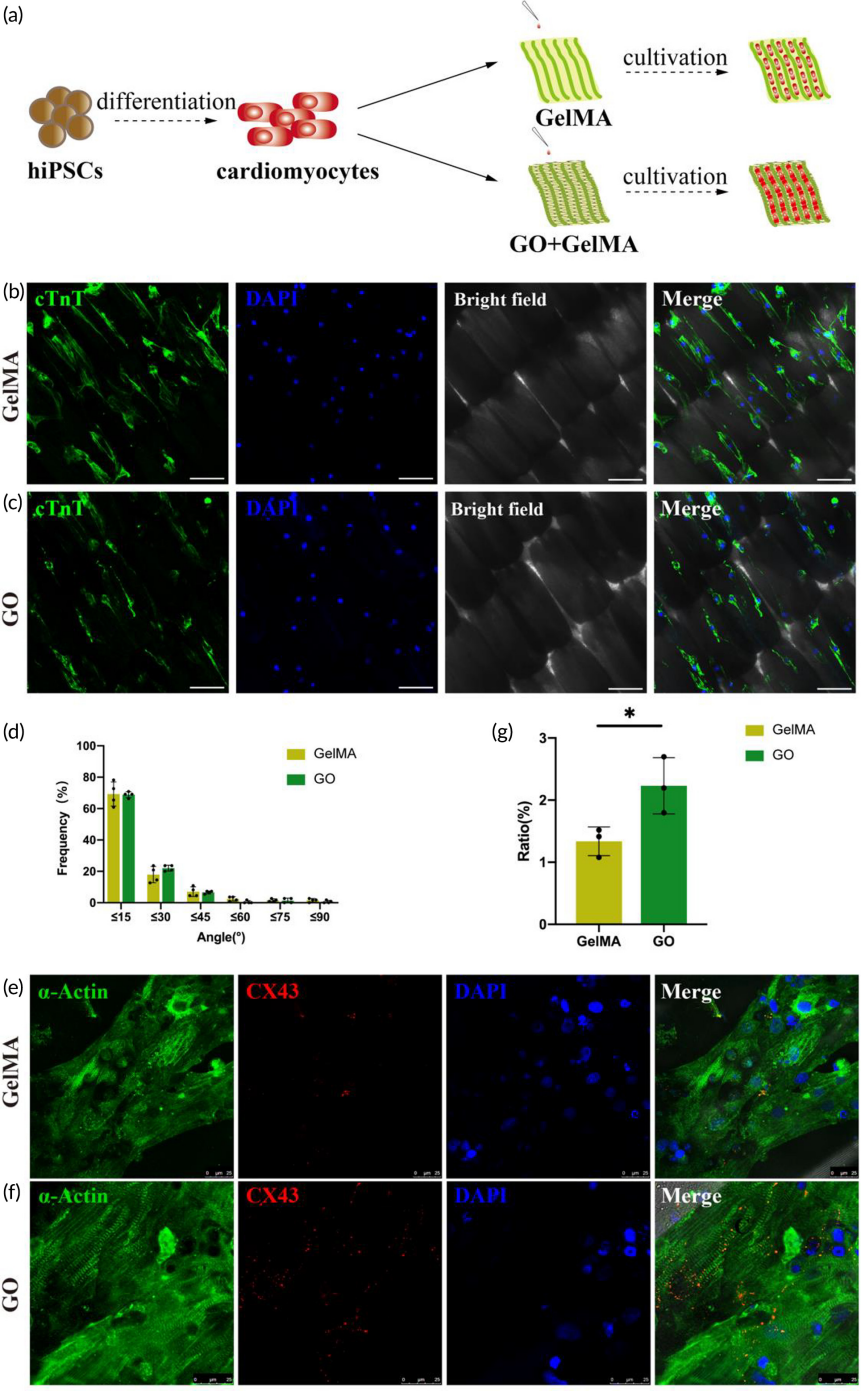
GO modified butterfly wings guide the directional alignment of hiPSC‐CMs and promote maturation. (a) Schematic diagram that represents the cellular seeding of hiPSC‐CMs on butterfly wings. (b) Immunofluorescence staining of hiPSC‐CMs arranged on the GelMA modified butterfly wing, scale bar, 100 μm, cTnT (green), DAPI (blue), brightfield. (c) Immunofluorescence staining of hiPSC‐CMs arranged on the GO modified butterfly wing, scale bar, 100 μm, cTnT (green), DAPI (blue), brightfield. (d) The effect of the topography of the butterfly wing on hiPSC‐CMs, GelMA (yellow), GO (green). (e) The immunofluorescence staining reveals the expression of CX43 in the hiPSC‐CMs on the GelMA modified butterfly wing. Scale bar, 25 μm. α‐Actin (green), CX43 (red), DAPI (blue). (f) The expression of CX43 in the hiPSC‐CMs on the GO modified butterfly wing via immunofluorescence. Scale bar, 25 μm. α‐Actin (green), CX43 (red), DAPI (blue). (g) Quantitative analysis of Cx43 expression in hiPSC‐CMs, GelMA (yellow), GO (green), * indicates the level of significance.

To test the feasibility of cultivating the hiPSC‐CMs on butterfly wings, we incubated hiPSC‐CMs on Day 15 of the differentiation protocol onto either GelMA or GO modified butterfly wings for 1 week (Figure 4a). iPSC‐CMs implanted on GelMA or GO modified butterfly wings were effectively guided by the groove structure of the butterfly wings (Figure 4b,c). We measured the angle between the hiPSC‐CMs and the nearby butterfly nanoridges to evaluate the alignment behavior of hiPSC‐CMs on GelMA and GO modified butterfly wings. According to our statistics, the GelMA and GO modified butterfly wings had 69.32 ± 7.66% and 68.86 ± 1.87% hiPSC‐CMs aligned within 15° orientation respectively. The percentage of the aligned hiPSC‐CMs cultivated on either GelMA or GO modified butterfly wings within 30° orientation reached 87.27 ± 2.68% and 90.91 ± 2.57%, respectively (Figure 4d). The groove structure has an obvious guiding effect on hiPSC‐CMs, which is consistent with the guiding effect previously reported with rodent cardiomyocytes.^1^ Previous studies have shown that directional arrangement can help to promote the unidirectional and synchronous contraction of cardiomyocytes in vitro, similar to that in vivo.^32^ According to several reports in the literature, increasing the conductivity of the scaffold can increase the electrical signal transduction between cardiomyocytes and promote the relative maturation of cardiomyocytes.^33^, ^34^, ^35^ In addition, GO has proven to be a promising nanomaterial that supports excellent conductivity.^36^ Interestingly, we found significant up‐regulation of the gap junction protein CX43, a major myocardial marker of maturation,^37^ in the hiPSC‐CMs cultivated on GO butterfly wings at 1‐week post‐cultivation (Figure 4e–g). This result implied that the addition of GO promotes the relative maturation of cardiomyocytes to a certain extent.

### 3.4 Engineering a conduction‐consistent cardiac patch with GO modified butterfly wings and hiPSC‐CMs

**FIGURE 5:**
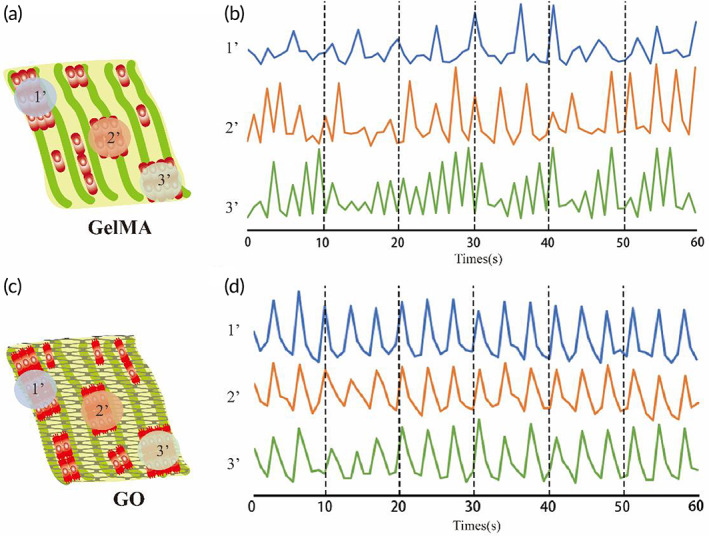
Simultaneous contraction of hiPSC‐CMs on butterfly wings. (a) Schematic diagram of three isolated hiPSC‐CMs clusters grown on GelMA modified butterfly wing for calcium imaging. (b) Representative calcium imaging traces of the three isolated hiPSC‐CMs clusters on GelMA modified butterfly wing, 1′ (blue trace), 2′ (orange trace), 3′ (green trace). (c) Schematic diagram of three isolated hiPSC‐CMs clusters grown on GO modified butterfly wing for calcium imaging. (d) Representative calcium imaging traces of the three isolated hiPSC‐CMs clusters on GelMA modified butterfly wing biosensor, 1′ (blue trace), 2′ (orange trace), 3′ (green trace).

We next investigated whether the addition of GO to butterfly wings could affect the contraction of the hiPSC‐CMs by improving the electrical signal conduction among cardiomyocytes. Here, we used calcium signals to correlate the contractile ability of cardiomyocytes. Specifically, we implemented rhod2 dye to stain the hiPSC‐CMs on the GelMA and GO modified butterfly wings and then capture the calcium signal changes of three random isolated hiPSC‐CM‐clusters using confocal microscopy under electrical stimulation. The acquired results indicated that the calcium signal traces of the three hiPSC‐CM‐clusters on the GelMA modified butterfly wings were irregular and asynchronous (Figure 5a,b; Video S2), while those on the GO modified butterfly wings were highly simultaneous (Figure 5c,d; Video S3). It is likely that the GO modified butterfly wings help promote the simultaneous calcium release of hiPSC‐CMs, enabling a more stable, regular, and simultaneous contraction of the cardiomyocytes. These data have implications for re‐purposing the GO modified butterfly wings to study the mechanisms and potential treatments of arrhythmia‐related and/or electrical conduction disorders.

### 3.5 GO modified butterfly wings promote the proliferation of hiPSC‐CPCs

**FIGURE 6:**
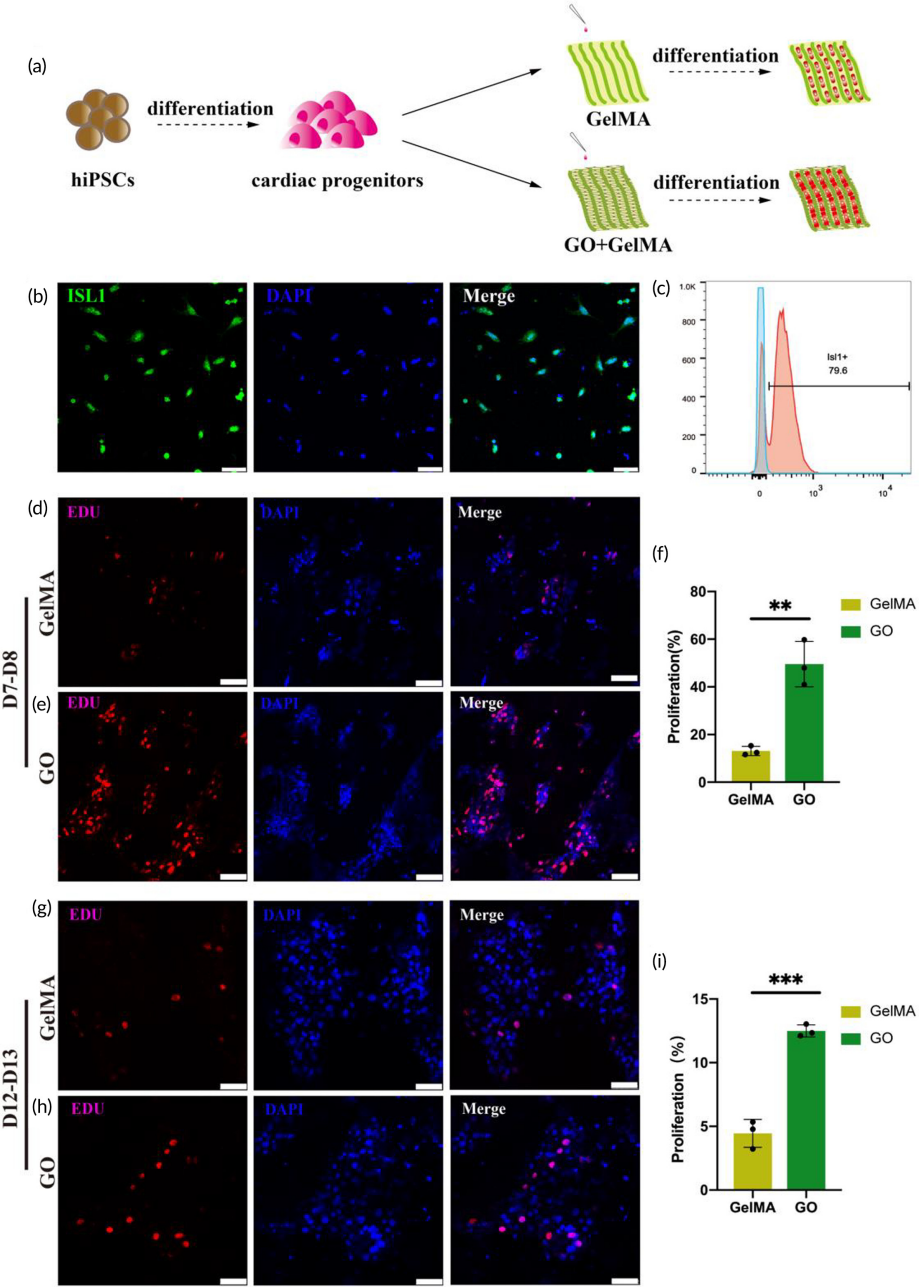
GO modified butterfly wings promote the proliferation of hiPSC‐CPCs. (a) Schematic diagram of seeding hiPSC‐CMs on butterfly wings. (b) The immunofluorescence staining of the hiPSC‐CPCs, prior to application on butterfly wings, scale bar, 50 μm. ISL1 (green), DAPI (blue). (c) Flow cytometry of ISL1 in the hiPSC‐CPCs on Day 6 prior to application on butterfly wings. (d) The early‐stage proliferation of hiPSC‐CPCs on the GelMA modified butterfly wings, scale bar, 50 μm, EDU (red), DAPI (blue). (e) The early‐stage proliferation of hiPSC‐CPCs on the GO modified butterfly wings, scale bar, 50 μm, EDU (red), DAPI (blue). (f) Statistics of early‐stage EDU expression in hiPSC‐CPCs in immunofluorescence, GelMA (yellow), GO (green). (g) The late‐stage proliferation of hiPSC‐CPCs on the GelMA modified butterfly wings, scale bar, 50 μm, EDU (red), DAPI (blue). (h) The late‐stage proliferation of hiPSC‐CPCs on the GO modified butterfly wings, scale bar, 50 μm, EDU (red), DAPI (blue). (i) Statistics of late‐stage EDU expression in hiPSC‐CPCs in immunofluorescence, GelMA (yellow), GO (green).

We next focused our efforts on investigating the ability of cells at earlier stages of the myocardial differentiation protocol to proliferate on butterfly wings. Of particular interest to us was the inoculation of cardiac progenitor cells (CPCs, differentiation time point Day 6) onto the butterfly wings (Figure 6a). In the case of the Day 6 cells, we first verified approximately 80% ISL1 expression by flow cytometry at this stage (Figure 6b,c), which was representative of a high‐quality cardiac differentiation, as previously reported.^9^ Next, to assess the effects GO modified butterfly wings had on cellular proliferation, we analyzed levels of incorporated EDU when cultivating CPCs on either GelMA or GO modified butterfly wings. We assessed cellular proliferation 24–48 h post‐implantation onto the butterfly wings, when the cells were on their 7th–8th day of differentiation (D7–D8); or following 6–7 days of cultivation on butterfly wings, when the cells were on their 12th–13th day of differentiation (D12–D13). We used the percentage of EDU‐positive cells to represent cellular proliferation rates. The cells seeded on GO modified butterfly wings measured 49.57 ± 9.51% (D7–D8) and 12.50 ± 0.47% (D12–D13) EDU‐positive respectively, compared with 13.12 ± 1.93% (D7–D8) and 4.45 ± 1.09% (D12–D13) on GelMA modified butterfly wings (Figure 6d–i). These data support previous findings that GO promotes the proliferation of hiPSC‐CPCs.^20^

### 3.6 GO modified butterfly wings promote the differentiation and maturation of hiPSC‐CPCs

We next sought to explore to what extent GO could alter the molecular and transcriptional profiles that drive cardiac differentiation and maturation. In our first set of experiments, hiPSC‐CPCs from Day 6 of the differentiation protocol were seeded onto either GelMA or GO modified butterfly wings and left to maturate for 4 days and 9 days post inoculation (named D10 and D15). In order to characterize the differences in functional gene networks between the different groups, we carried out bulk RNA‐Sequencing analysis on the D10 and D15 cells, and compared the transcriptional profiles to those from the hiPSC‐CPCs (named D6), prior to the implantation on butterfly wings.

Principal component analysis showed that the D10 and D15 cells on GelMA or GO were clustered at different time points after differentiation (Figure S2a). We next analyzed the correlation of all samples and mapped out the differentially expressed genes (DEGs) between samples (Figure S2b,c).

**FIGURE 7:**
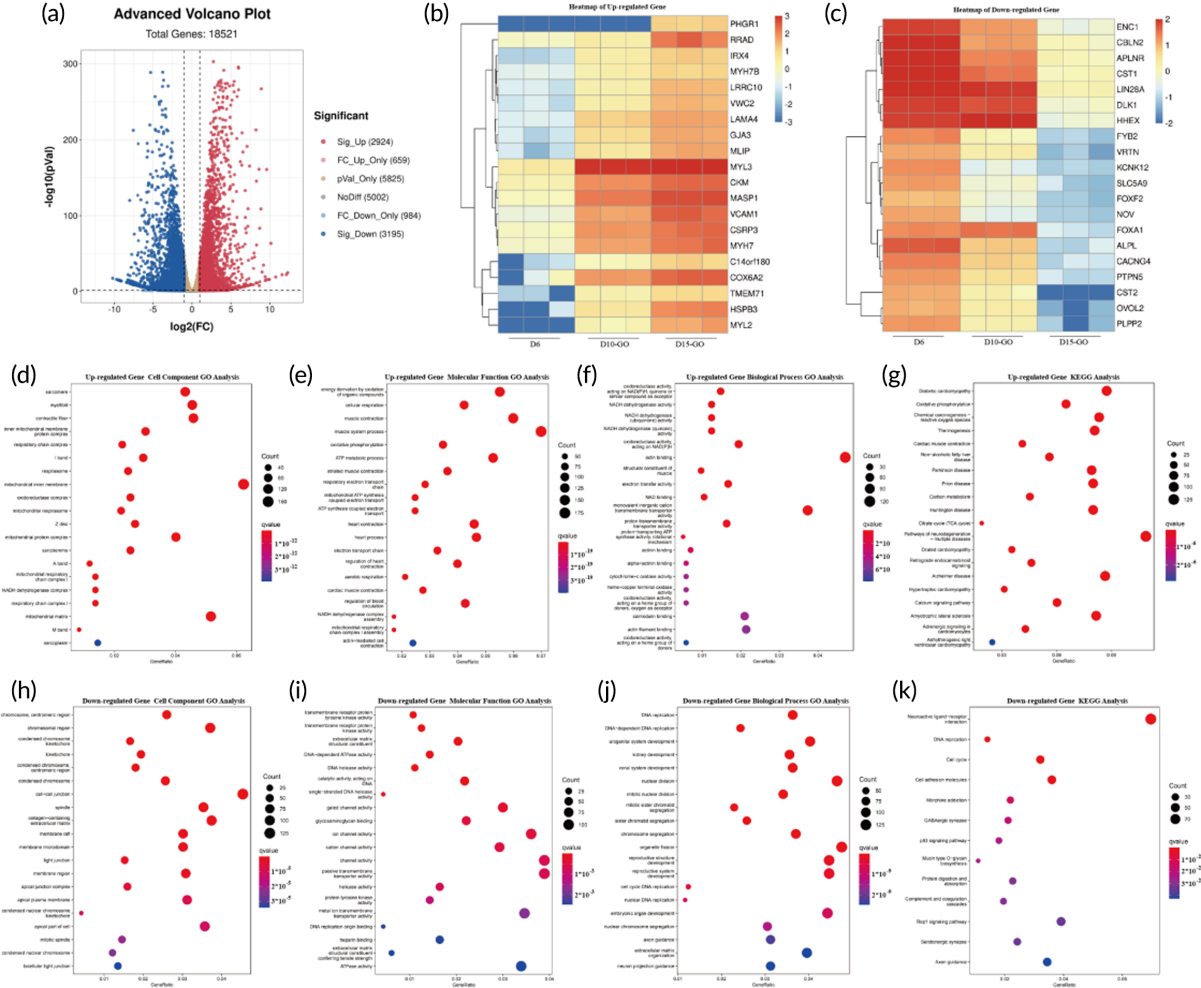
Differential gene expression profiles of hiPSC‐CPCs during differentiation and maturation on the GO modified butterfly wings. (a) Volcano plot depicting differentially expressed genes (DEGs) from D6 hiPSC‐CPCs and D15 hiPSC‐CMs on GO modified butterfly wings. The *x* axis represents the fold change of the difference after log_2_ conversion, and the *y* axis represents the significance value after −log_10_ conversion. Red (up‐regulated DEGs), blue (down‐regulated DEGs), gray (non‐DEGs), pink (fold‐change up‐regulated only), cerulean (fold‐change down‐regulated only), orange (significance value only). (b, c) Heatmap comparing relative gene expression patterns of representative DEGs between hiPSC‐CPCs and hiPSC‐CMs on GO modified butterfly wings. Blue represents low intensity expression, and red represents high intensity expression. The expression is showed after −log_10_ conversion. (d–f) Up‐regulated gene oncology enrichment analysis between hiPSC‐CPCs and hiPSC‐CMs on GO modified butterfly wings on Day 15, including cellular component (d), molecular function (e), biology process (f). (g) Up‐regulated KEGG analysis between hiPSC‐CPCs and hiPSC‐CMs on GO modified butterfly wings. (h–j) Down‐regulated gene oncology enrichment analysis between hiPSC‐CPCs and hiPSC‐CMs on GO modified butterfly wings, including cellular component (h), molecular function (i), biology process (j). (k) Down‐regulated KEGG analysis between hiPSC‐CPCs and hiPSC‐CMs on GO modified butterfly wings.

Next, to elucidate specifically the transcriptional differences between the undifferentiated D6 control cells with the D15 cells differentiated on either the GO or GelMA modified butterfly wings respectively, we performed enrichment analysis of the differentially expressed genes and found that the genes of the two groups changed greatly in the process of differentiation and maturation (Figure 7; S3). First, we analyzed and compared the RNA seq data of CPCs cultivated on the GO modified butterfly wings at different time points (D6 vs. D10 vs. D15). We found 2924 significantly up‐regulated genes and 3195 significantly down‐regulated genes on D15 compared with D6 (Figure 7a). Heat map analyses revealed the top 20 over and under‐regulated DEGs (Figure 7b,c). The top 20 up‐regulated DEGs include well‐known genes such as MYH7, GJA3 (CX43), and MYL2 that is corresponding with the myocardial differentiation and maturation (Figure 7b). We enriched and analyzed these differential genes of D15 hiPSC‐CMs by Gene ontology next. The up‐regulated DEGs are mainly enriched in go items such as sarcomere, myofibril, and contractile fiber at the cellular component level (Figure 7d). With regards to molecular function, the DEGs are mainly enriched in go items such as energy derivation by oxidation of organic compounds, cellular respiration, and muscle contraction (Figure 7e). On a biological processing level, the DEGs are mainly enriched in go items such as oxidoreductase activity, NADH dehydrogenase activity, and actin binding which are all related to maturation of muscle fiber and energy metabolism (Figure 7f). Further data analysis with KEGG databases map DEGs related to diabetic cardiomyopathy, cardiac muscle contraction, and carbon metabolism (Figure 7g). The down‐regulated DEGs cluster with cell cycle and DNA replication, which is consistent with the process of myocardial differentiation (Figure 7h–k).

Next, we analyzed and compared the RNA seq datasets between the GelMA modified butterfly wings at different time point, namely the D6 undifferentiated cells versus D10 and D15 differentiated cells. There were a total of 2807 up‐regulated genes and 3128 down‐regulated genes when comparing D15 cultivated cells on GelMA modified butterfly wings with D6 hiPSC‐CPCs (Figure S3a). However, the gene ontology analysis results stemming from the GelMA group were similar to those obtained from the GO group, including up‐regulating of genes corresponding with the myocardial differentiation and maturation like MYH7, GJA3 (CX43), and MYL2 (Figure S3b–k). The above results suggested that hiPSC‐CPCs can successfully differentiate into cardiomyocytes on both GO and GelMA modified butterfly wings.

**FIGURE 8:**
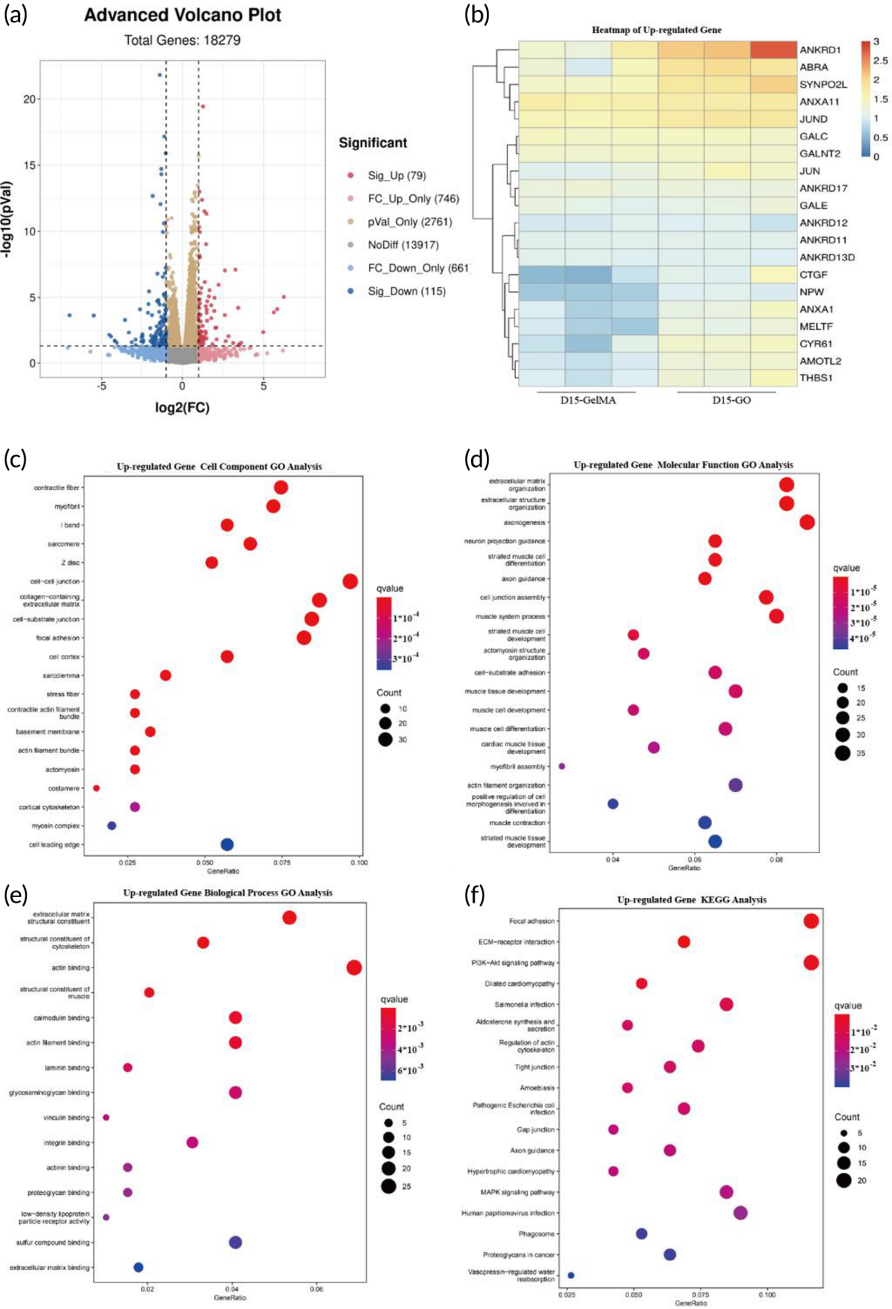
Differential gene expression profiles from D15 hiPSC‐CMs inoculated on GelMA versus GO modified butterfly wings. (a) The volcano plot depicts differentially expressed genes (DEGs) between the hiPSC‐CMs on GelMA or GO modified butterfly wings. The *x* axis represents the fold change of the difference after log_2_ conversion, and the *y* axis represents the significance value after −log_10_ conversion. Red (up‐regulated DEGs), blue (down‐regulated DEGs), gray (non‐DEGs), pink (fold‐change up‐regulated only), cerulean (fold‐change down‐regulated only), orange (significance value only). (b) Heatmap comparing relative gene expression patterns of representative up‐regulated DEGs of hiPSC‐CMs on GelMA and GO modified butterfly wings. Blue represents low intensity expression, and red represents high intensity expression. The expression is showed after −log_10_ conversion. (c–e) Up‐regulated gene oncology enrichment analysis results of hiPSC‐CMs on GelMA and GO modified butterfly wings, including cellular component (c), molecular function (d), biology process (e). (f) Up‐regulated KEGG analysis results of hiPSC‐CMs on GelMA and GO modified butterfly wings.

In order to further explore the promoting effect of GO on the differentiation of hiPSC‐CPCs, we conducted a further comparison of the DEGs between the gene sets from cells applied on the two kinds of modified butterfly wings. We mapped volcano plots based on gene signatures stemming from the DEGs of the D10 and D15 cells, separately (Figure 8a; Figure S4a). When analyzing the transcriptional profiles of Day 15 cells having been inoculated on either GelMA or GO butterfly wings, we found 79 up‐regulated genes and 115 down‐regulated genes between the two groups. As performed previously, we again analyzed the significant up‐regulated DEGs by Gene ontology and pick up the top 20 up‐regulated genes on D15 (Figure 8b), and on D10 (Figure S4b). Regarding the cell component level, the DEGs were mainly enriched in the GO group for contractile fibers, z disc, and cell–cell junctions (Figure 8c). Regarding transcripts supporting molecular function, the DEGs were mainly enriched in go items such as extracellular matrix organization and muscle system processes in the GO group (Figure 8d). On the biological process level, the DEGs were mainly enriched in go items such as structural constituent of cytoskeleton, calmodulin binding, and actin binding in GO group (Figure 8e). Several of these enriched gene clusters were related to maturated cardiomyocytes. The KEGG analysis found that the DEGs were mainly enriched within the PI3K‐Akt signaling pathway and gap junction genes (Figure 8f). These genes included classical maturity related genes PPARGC1A, SLC8A1, and PLN, as well as genes previously reported related to myocardial differentiation such as LAMA2, ERBB4, BVES, and NCA1M.^38^, ^39^, ^40^, ^41^, ^42^ Those results suggested that hiPSC‐CPCs can differentiate into cardiomyocytes better on GO modified butterfly wings than on GelMA modified butterfly wings.

## 4 DISCUSSION

Herein, our research innovatively applied rGO to modify butterfly wings in order to emulate two important characteristics of the natural heart: (1) structural orientation and arrangement of the myocardial cells; (2) consistent conduction of signaling between the heart cells.^43^, ^44^, ^45^, ^46^ Together, we successfully constructed myocardial tissue patches with consistent conduction, providing a new model for the study of electrical cardiac properties. During this process, the special nanostructure of butterfly wings facilitated the parallel orientation of hiPSC‐CMs, while GO further facilitated the relative maturation and the conduction consistency of hiPSC‐CMs. At the same time, we showed that GO modified butterfly wings better promote the differentiation and maturation of cardiac progenitors through functional assay readouts and omics analysis. These results demonstrate a novel system for hiPSC‐CMs maturation and in addition showcase a cell‐based cardiac development model through the application of culturing hiPSC‐CPCs on GO‐enriched butterfly wings.

The construction of a patch model with conduction consistency better simulates the electrophysiological characteristics of the heart, which is very important for the study of cardiac diseases and drug screening. To achieve this goal, a material with good conduction consistency is required. Butterfly wings are natural materials, whose surface contains special nanostructures, allowing for directional adhesion functions and directional transport properties.^2^, ^3^, ^4^, ^5^, ^6^, ^47^, ^48^ Butterfly wings are made of chitin structures, which play an important role in the field of tissue engineering due to their large surface area to volume ratios and high porosities.^6^, ^49^, ^50^ At the same time, butterfly wings may have subtle, individual differences that could lead to variations within the system. Apart from these differences and potential environmental protection problems, nature has provided the most suitable structure for alignment and conduction consistency that no artificial level of technology has simulated to date.^1^ Perhaps with the development of science and technology, substitute materials can be manufactured in the future. Furthermore, the butterfly wings are easy to obtain, low cost, and easy to prepare and modify. Taken together, butterfly wings are ideal scaffolds for improving the electrical conductivity of iPSC‐CMs, but require further modifications to employ an optimized system.

GO is an important conductive material, composed of a single layer of carbon atoms, arranged in a honeycomb lattice shape.^51^ GO has recently attracted the interest of many researchers due to the large surface area, excellent electrical and thermal conductivity, strong surface chemistry, and excellent mechanical strength.^17^ The addition of GO to non‐conductive substances has been shown to significantly enhance electrical conductivity.^20^ Moreover, previous studies conveyed GO as an important compound for improved adhesion, growth, proliferation, and differentiation of stem cells,^16^, ^18^, ^19^ which indicates that GO plays an important role in tissue engineering research. Several previous studies have shown the therapeutic effects of GelMA and/or GO on myocardial infarction, but most of these studies do not provide mechanistic data and instead focus on the impact of GO on cardiomyocytes.^7^, ^52^, ^53^ In our study we comprehensively considered the importance of the structural characteristics of the myocardium and the consistent electrical conduction that is required to maintain cardiac function. On the basis of the results of many studies that have highlighted the directional arrangement of cardiac cells to promote the maturation of cardiomyocytes,^15^, ^54^, ^55^ we added the role of conductive substances to build a more appropriate cardiac tissue patch. In this study, we used GO to modify butterfly wings. Different from previous studies, whose aim was to build a platform for cardiomyocytes detection,^1^ the purpose of our study was to explore the effects of conductive substances and directional arrangement on the structure and function of cardiomyocytes. The treatment of butterfly wings with GO resulted in improved cellular guidance and directional arrangement of hiPSC‐CMs, as well as enhanced connectivity and electrical conductivity of the hiPSC‐CMs (Figures 4 and 5). We also demonstrated that GO modified butterfly wings promoted the proliferation of hiPSC‐CPCs, as indicated by the increased presence of EDU staining at both the early and late stage differentiation windows (Figure 6d–i). Furthermore, the differentiation and maturation of hiPSC‐CPCs appeared far more capable on the GO enriched butterfly wings than the GelMA treated butterfly wings, based on the increased stainings of maturation marker Cx43 (Figure 4e–g), improved electrical conductivity (Figure 5) and a number of significantly upregulated transcripts that relate to structural and metabolic maturity (Figures 7 and 8). We speculate the major mechanisms of action the GO modified butterfly wings have on the aforementioned differentiation and maturation of hiPSC‐CPCs is as follows: (1) The chemical structure or properties of GO have an impact on the differentiation of hiPSC‐CPCs and the maturation of hiPSC‐CMs, and further maturation of hiPSC‐CMs increases the gap junctions between cells, thereby improving the electrical conduction consistency between hiPSC‐CMs. (2) Modification of the butterfly wings with GO increases their electrical conductivity, which improves electrical conduction consistency between the cells seeded on them. And the consistent electrical signal changes among cells promote the hiPSC‐CPCs differentiation into hiPSC‐CMs and the maturation of hiPSC‐CMs. Further research is needed to more greatly explore the molecular and cellular underpinnings driving these results.

In this study, we generated a conduction‐consistent patch using GO‐treated butterfly wings with hiPSC‐CMs, which could be further established for personalized heart disease research.^56^, ^57^, ^58^, ^59^ In our follow‐up studies, we plan to employ our system for the disease modeling of inherited cardiomyopathies and drug discovery research. The system developed by our team could also be employed to study the in vitro differentiation process of hiPSCs and the development of early‐stage heart progenitors for congenital disease modeling. We believe the construction of the conduction‐consistent in vitro cardiac patch reported herein has a wide range of application value.

## 5 CONCLUSION

In summary, we inoculated hiPSC‐CMs on GO modified butterfly wings to construct a cardiac muscle patch capable of maintaining conduction consistency. Future research studies aim to use this patch to explore the details of the complex cardiac conduction system and the mechanisms of arrhythmia. The derivation of a cardiac patch on GO‐modified butterfly wings can also be employed to monitor or study the effects of cardiovascular drugs and how they interfere with electrical conduction. Furthermore, the capability for GO to promote differentiation and maturation of hiPSC‐CPCs will allow researchers to build biological models to study the cellular components of cardiac development. We believe the establishment of this cardiac model system offers a wide range of biomedical application and value.

## AUTHOR CONTRIBUTIONS

**Yao Tan:** Data curation (equal); formal analysis (equal); methodology (equal); writing – original draft (equal); writing – review and editing (equal). **Tingting Lu:** Data curation (equal); formal analysis (equal); methodology (equal); writing – review and editing (equal). **Ying Chen:** Data curation (equal); methodology (equal); writing – original draft (equal); writing – review and editing (equal). **Nevin Witman:** Writing – review and editing (supporting). **Bingqian Yan:** Methodology (supporting); writing – review and editing (supporting). **Li Yang:** Data curation (supporting); methodology (supporting); writing – review and editing (supporting). **Minglu Liu:** Formal analysis (supporting); methodology (supporting). **Yiqi Gong:** Formal analysis (supporting); methodology (supporting). **Xuefeng Ai:** Methodology (supporting). **Runjiao Luo:** Methodology (supporting). **Huijing Wang:** Data curation (supporting); methodology (supporting). **Wei Wang:** Formal analysis (supporting); funding acquisition (equal); methodology (supporting); writing – review and editing (supporting). **Wei Fu:** Funding acquisition (lead); investigation (lead); methodology (equal); writing – review and editing (equal).

## CONFLICT OF INTEREST STATEMENT

The authors declare no conflicts of interest.

## Supporting information


**Figure S1.** The molecular validation of human induced pluripotent stem cells. (a–d) Representative immunostaining and microscopy images of iPSC cell clusters. (A) Immunofluorescence staining of OCT4. (b) Immunofluorescence staining of TRA‐1‐60. (c) Immunofluorescence staining of SOX2. (d) Immunofluorescence staining of NANOG. Scale bar, 100 μm.
**Figure S2.** General overview of the RNA‐seq samples. (a) Principal component analysis. Each point represents a group of cells in the same condition. (b) Correlation analysis of the samples. (c) Total differentially expressed genes. Red (up‐regulated DEGs), blue (down‐regulated DEGs) and *y* axis represents the number of differential genes.
**Figure S3.** Differential gene expression profiles of hiPSC‐CPCs during differentiation and maturation on the GelMA modified butterfly wings. (a) The volcano plot of differentially expressed genes (DEGs) between D6 hiPSC‐CPCs and D15 hiPSC‐CMs on GelMA modified butterfly wings. The *x* axis represents the fold change of the difference after log_2_ conversion and the *y* axis represents the significance value after −log_10_ conversion. Red (up‐regulated DEGs), blue (down‐regulated DEGs), gray (non‐DEGs), pink (fold‐change up‐regulated only), cerulean (fold‐change down‐regulated only), orange (significance value only). (b, c) Heatmap comparing relative gene expression patterns of representative DEGs between D6 hiPSC‐CPCs as well as D10 and D15 hiPSC‐CMs on GelMA modified butterfly wings. Blue represents low intensity expression, and red represents high intensity expression. The expression is shown after −log_10_ conversion. (d–f) Up‐regulated gene oncology enrichment analysis between hiPSC‐CPCs and hiPSC‐CMs on GelMA modified butterfly wings, including cellular component (d), molecular function (e), biology process (f). (g) Results depicting the up‐regulated KEGG analysis from the cells on GO modified butterfly wings. (h–j) Down‐regulated gene oncology enrichment analysis between the hiPSC‐CPCs and hiPSC‐CMs on GelMA modified butterfly wings, including cellular component (h), molecular function (i), biology process (j). (h) Down‐regulated KEGG analysis between the hiPSC‐CPCs and hiPSC‐CMs on GelMA modified butterfly wings.
**Figure S4.** Differential gene expression profiles of D10 hiPSC‐CMs between GelMA and GO modified butterfly wings. (a) Volcano plot of differentially expressed genes (DEGs) from hiPSC‐CPCs and hiPSC‐CMs on GelMA or GO modified butterfly wings on Day 10. The *x* axis represents the fold change of the difference after log_2_ conversion, and the *y* axis represents the significance value after −log_10_ conversion. Red (up‐regulated DEGs), blue (down‐regulated DEGs), gray (non‐DEGs), pink (fold‐change up‐regulated only), cerulean (fold‐change down‐regulated only), orange (significance value only). (b) Heatmap comparing relative gene expression patterns of representative up‐regulated DEGs of hiPSC‐CMs on GelMA and GO modified butterfly wings on Day 10. Blue represents low intensity expression, and red represents high intensity expression. The expression is showed after −log_10_ conversion.

**Video S1.** Autonomous beating of hiPSC‐CMs on 10th day of differentiation.

**Video S2.** Calcium imaging of the hiPSC‐CMs clusters on GelMA butterfly wings (6× speed).

**Video S3.** Calcium imaging of the hiPSC‐CMs clusters on GO butterfly wings (6× speed).

## Data Availability

The data that support the findings of this study are available in the Supporting Information of this article.
