# Supplementary material for: Engineering a conduction‐consistent cardiac patch with graphene oxide modified butterfly wings and human pluripotent stem cell‐derived cardiomyocytes
Source: Bioeng Transl Med. 2023 Apr 14;8(3):e10522. doi: 10.1002/btm2.10522 (PMC10189447; doi:10.1002/btm2.10522)
Supplement: Supplementary file 1 — Figure S1. The molecular validation of human induced pluripotent stem cells. (a–d) Representative immunostaining and microscopy images of iPSC cell clusters. (A) Immunofluorescence staining of OCT4. (b) Immunofluorescence staining of TRA‐1‐60. (c) Immunofluorescence staining of SOX2. (d) Immunofluorescence staining of NANOG. Scale bar, 100 μm. Figure S2. General overview of the RNA‐seq samples. (a) Principal component analysis. Each point represents a group of cells in the same condition. (b) Correlation analysis of the samples. (c) Total differentially expressed genes. Red (up‐regulated DEGs), blue (down‐regulated DEGs) and y axis represents the number of differential genes. Figure S3. Differential gene expression profiles of hiPSC‐CPCs during differentiation and maturation on the GelMA modified butterfly wings. (a) The volcano plot of differentially expressed genes (DEGs) between D6 hiPSC‐CPCs and D15 hiPSC‐CMs on GelMA modified butterfly wings. The x axis represents the fold change of the difference after log2 conversion and the y axis represents the significance value after −log10 conversion. Red (up‐regulated DEGs), blue (down‐regulated DEGs), gray (non‐DEGs), pink (fold‐change up‐regulated only), cerulean (fold‐change down‐regulated only), orange (significance value only). (b, c) Heatmap comparing relative gene expression patterns of representative DEGs between D6 hiPSC‐CPCs as well as D10 and D15 hiPSC‐CMs on GelMA modified butterfly wings. Blue represents low intensity expression, and red represents high intensity expression. The expression is shown after −log10 conversion. (d–f) Up‐regulated gene oncology enrichment analysis between hiPSC‐CPCs and hiPSC‐CMs on GelMA modified butterfly wings, including cellular component (d), molecular function (e), biology process (f). (g) Results depicting the up‐regulated KEGG analysis from the cells on GO modified butterfly wings. (h–j) Down‐regulated gene oncology enrichment analysis between the hiPSC‐CPCs and hiPSC‐CMs [file BTM2-8-e10522-s002.docx]

**Supporting Figures**


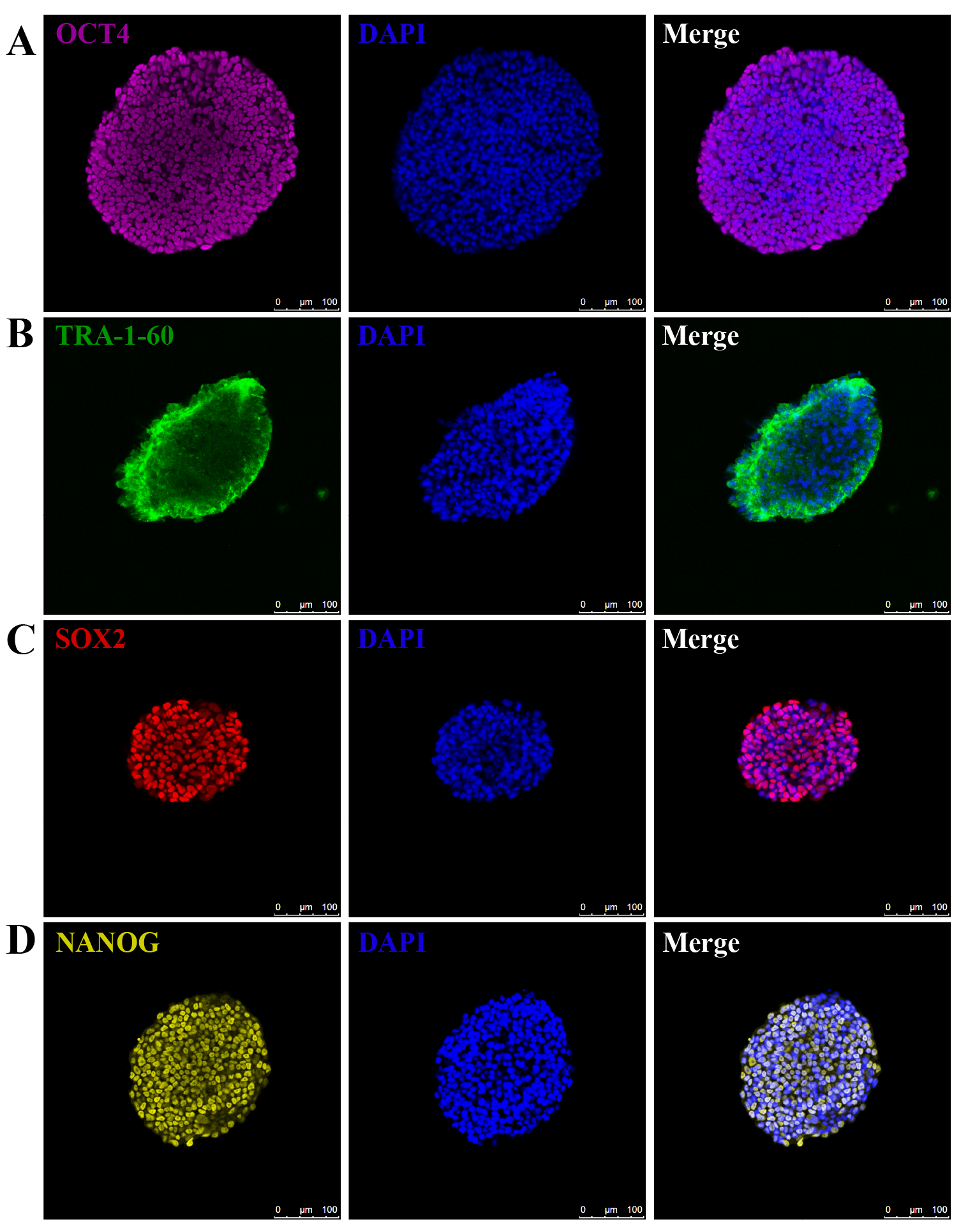


**Figure S1. The molecular validation of human induced pluripotent stem cells.** (A-D) Representative immuno-stainings and microscopy images of iPSC cell clusters. (A) Immunofluorescence staining of OCT4. (B) Immunofluorescence staining of TRA-1-60. (C) Immunofluorescence staining of SOX2. (D) Immunofluorescence staining of NANONG. Scale bar, 100 μm.


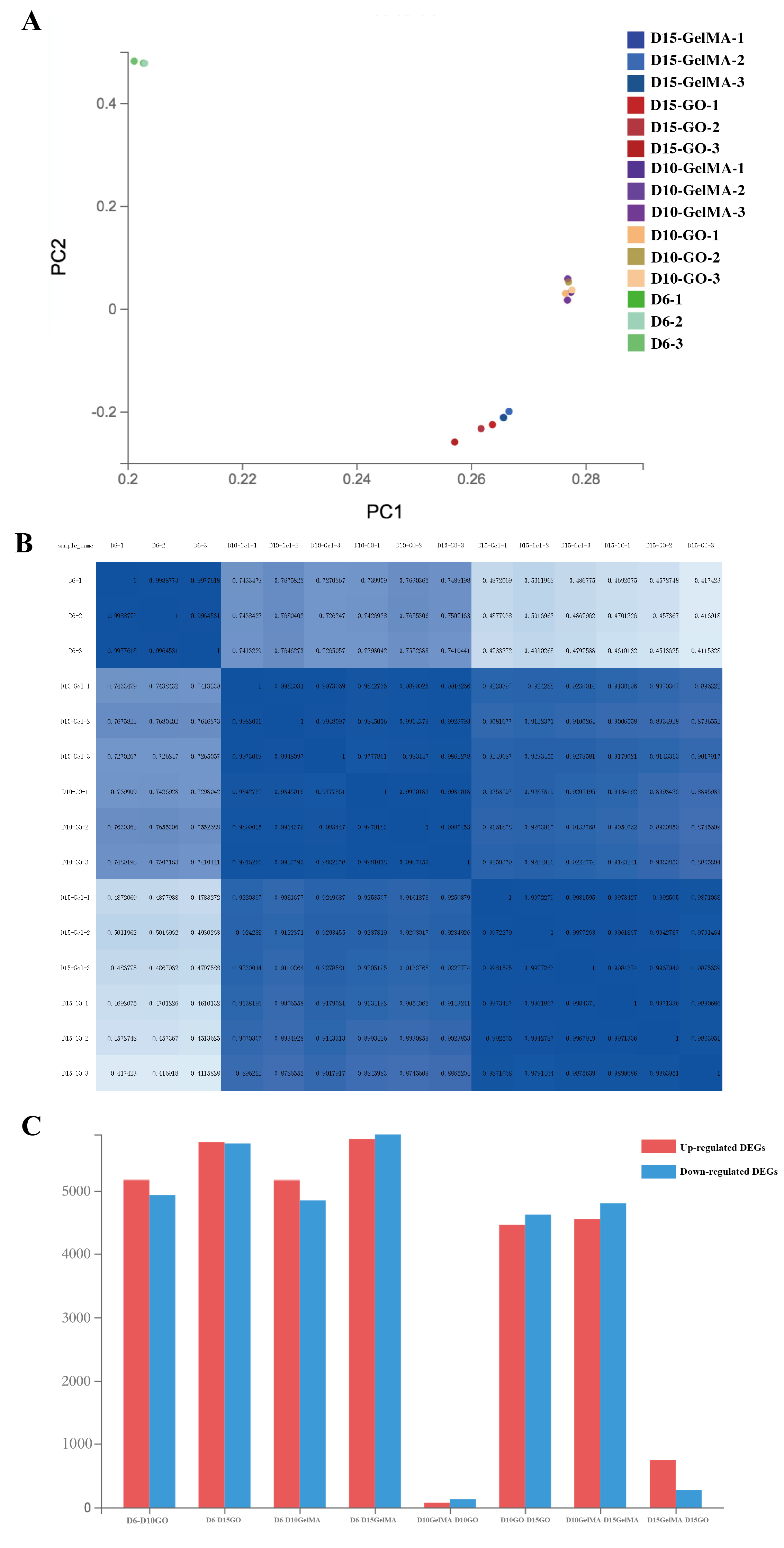


**Figure S2. General overview of the RNA-seq samples.** (A) Principal component analysis. Each point Represents a group of cells in the same condition. (B) Correlation analysis of the samples. (C) Total differentially expressed genes. Red (up-regulated DEGs), blue (down-regulated DEGs) and Y-axis represents the number of differential genes.


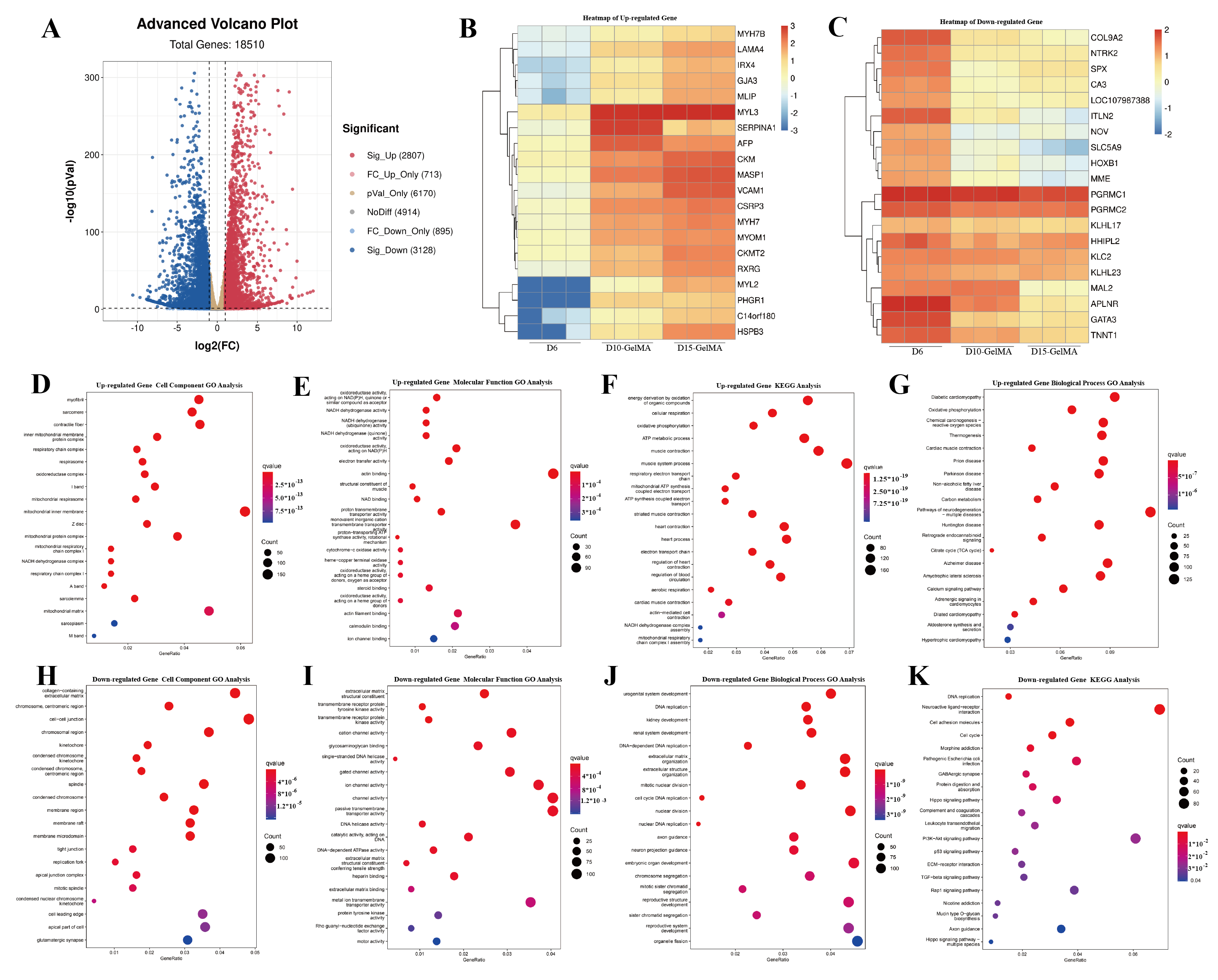


**Figure S3. Differential gene expression profiles of hiPSC-CPCs during differentiation and maturation on the GelMA modified butterfly wings.** (A) The volcano plot of differentially expressed genes (DEGs) between D6 hiPSC-CPCs and D15 hiPSC-CMs on GelMA modified butterfly wings. The X-axis represents the fold change of the difference after log2 conversion and the Y-axis represents the significance value after −log10 conversion. Red (up-regulated DEGs), blue (down-regulated DEGs), gray(non-DEGs), pink (fold-change up-regulated only), cerulean (fold-change down-regulated only), orange (significance value only). (B-C) Heatmap comparing relative gene expression patterns of representative DEGs between D6 hiPSC-CPCs as well as D10 and D15 hiPSC-CMs on GelMA modified butterfly wings. Blue represents low intensity expression, and red represents high intensity expression. The expression is shown after −log10 conversion. (D-F) Up-regulated gene oncology enrichment analysis between hiPSC-CPCs and hiPSC-CMs on GelMA modified butterfly wings, including cellular component(D), molecular function(E), biology Process(F). (G) Results depicting the up-regulated KEGG analysis from the cells on GO modified butterfly wings. (H-J) Down-regulated gene oncology enrichment analysis between the hiPSC-CPCs and hiPSC-CMs on GelMA modified butterfly wings, including cellular component(H), molecular function(I), biology Process(J). (H) Down-regulated KEGG analysis between the hiPSC-CPCs and hiPSC-CMs on GelMA modified butterfly wings.


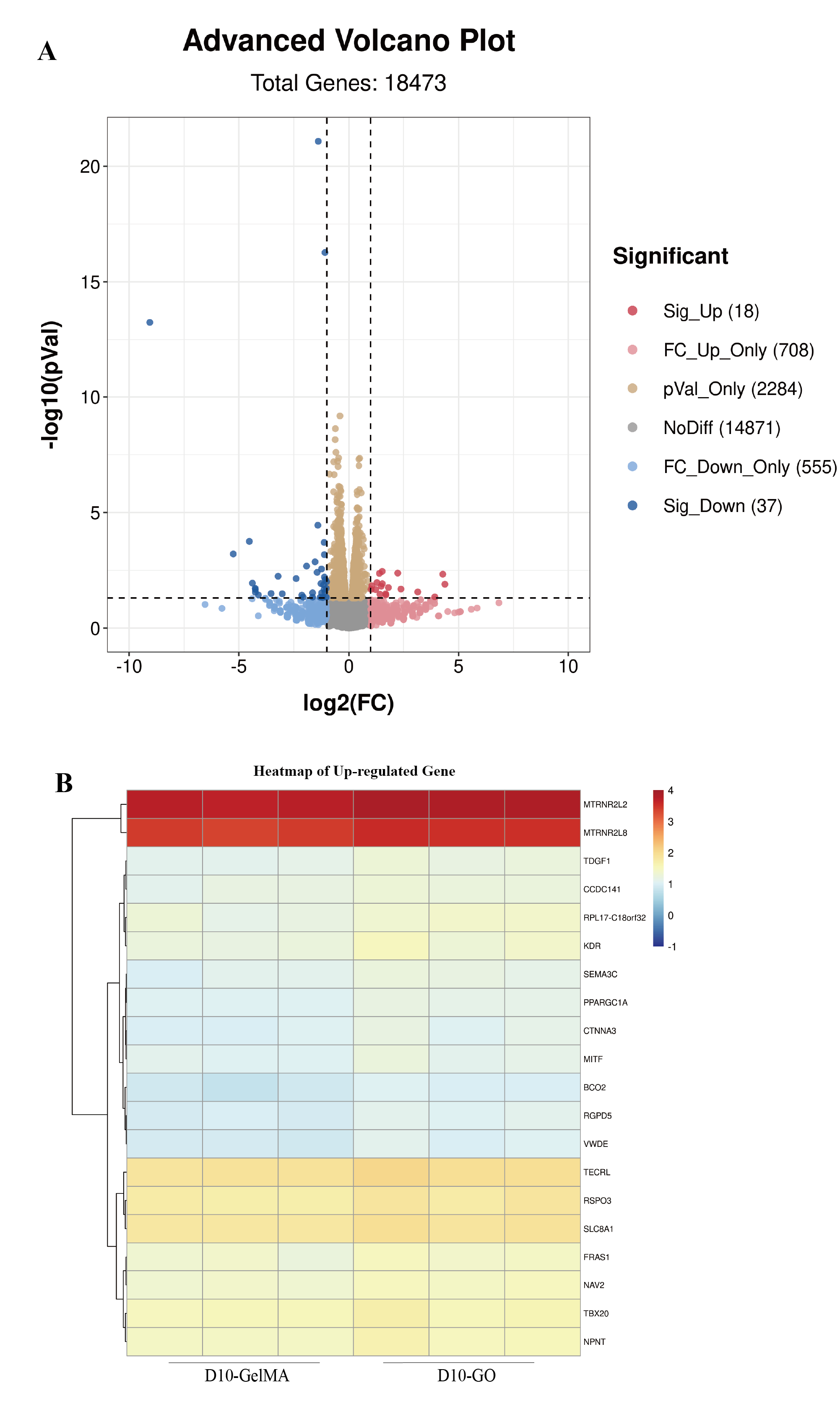


**Figure S4. Differential gene expression profiles of D10 hiPSC-CMs between GelMA and GO modified butterfly wings.** (A) Volcano plot of differentially expressed genes (DEGs) from hiPSC-CPCs and hiPSC-CMs on GelMA or GO modified butterfly wings on day 10. The X-axis represents the fold change of the difference after log2 conversion, and the Y-axis represents the significance value after −log10 conversion. Red (up-regulated DEGs), blue (down-regulated DEGs), gray (non-DEGs), pink (fold-change up-regulated only), cerulean (fold-change down-regulated only), orange (significance value only). (B) Heatmap comparing relative gene expression patterns of representative up-regulated DEGs of hiPSC-CMs on GelMA and GO modified butterfly wings on day 10. Blue represents low intensity expression, and red represents high intensity expression. The expression is showed after −log10 conversion.
